# Changes in physical activity across pregnancy among Chinese women: a longitudinal cohort study

**DOI:** 10.1186/s12905-021-01377-3

**Published:** 2021-06-06

**Authors:** Yan Lü, Yahui Feng, Shuai Ma, Yu Jiang, Liangkun Ma

**Affiliations:** 1 Department of Obstetrics and Gynecology, Peking Union Medical College Hospital, Chinese Academy of Medical Science and Peking Union Medical College, No. 1 Shuaifuyuan Wangfujing Dongcheng District, 100730 Beijing, People’s Republic of China; 2 School of Public Health, Chinese Academy of Medical Science and Peking Union Medical College, Beijing, China

**Keywords:** Physical activity, Pregnancy, Change across pregnancy, Chinese women

## Abstract

**Background:**

Sufficient physical activity (PA) during pregnancy is beneficial for a woman’s health; however, the PA levels of Chinese women at different pregnancy stages are not clear. The aim of our study was to investigate PA changes during pregnancy and the association of population characteristics with PA change among Chinese women.

**Methods:**

Data were obtained from 2485 participants who were enrolled in the multicentre prospective Chinese Pregnant Women Cohort Study. PA level was assessed in early pregnancy (mean = 10, 5–13 weeks of gestation) and again in mid-to-late pregnancy (mean = 32, 24–30 weeks of gestation) using the International Physical Activity Questionnaire short form (IPAQ-SF). Sufficient PA (≥ 600 MET min/week) in early pregnancy and insufficient PA in mid-to-late pregnancy indicated decreasing PA. Insufficient PA in early pregnancy and sufficient PA in mid-to-late pregnancy indicated increasing PA. The associations between demographic, pregnancy and health characteristics and PA changes were examined by multivariable logistic regression.

**Results:**

Total energy expenditure for PA increased significantly from early (median = 396 MET min/week) to mid-to-late pregnancy (median = 813 MET min/week) (*P* < 0.001), and 55.25% of the participants eventually had sufficient PA. Walking was the dominant form of PA. Women with sufficient PA levels in early pregnancy were more likely to have sufficient PA in mid-to-late pregnancy (OR 1.897, 95% CI 1.583–2.274). Women in West China and those in Central China were most and least likely, respectively, to have increasing PA (OR 1.387, 95% CI 1.078–1.783 vs. OR 0.721, 95% CI 0.562–0.925). Smoking was inversely associated with increasing PA (OR 0.480, 95% CI 0.242–0.955). Women with higher educational levels were less likely to have decreasing PA (OR 0.662, 95% CI 0.442–0.991).

**Conclusions:**

PA increased as pregnancy progressed, and walking was the dominant form of PA among Chinese women. Further research is needed to better understand correlates of PA change.

**Supplementary Information:**

The online version contains supplementary material available at 10.1186/s12905-021-01377-3.

## Background

Physical activity (PA) is bodily movement produced by skeletal muscles that requires energy expenditure, including activities undertaken while working, playing, carrying out household chores, travelling, and engaging in recreational activities [1]. PA during pregnancy is not only safe [1, 2] but also helpful in improving physical health, controlling weight, alleviating back pain, accelerating postpartum recovery and reducing the risk of gestational diabetes, preeclampsia and operative delivery, as well as relieving depression and anxiety [3–16]. To achieve good health, the World Health Organization (WHO) recommends that adults engage in at least 150 min of moderate-intensity PA throughout the week or an equivalent form of PA that achieves at least 600 metabolic equivalent task (MET) min/week [1, 17]. Pregnant women are advised to maintain the same level of PA as non-pregnant women [18–20].

Successful intervention for PA requires understanding PA level across pregnancy and its correlates. Previous studies have shown that PA tends to decrease during pregnancy compared with the prepregnancy period [21, 22]. The proportion of pregnant women achieving the recommended PA level varies widely from 11–66% worldwide, and this wide range is mainly due to different population characteristics and assessment times [22–24]. Few studies have investigated changes in PA over the course of pregnancy, and the results have varied [13, 25, 26]. The results of studies on the association of population characteristics (e.g., demographic, pregnancy and health characteristics) with PA during pregnancy vary [26–29]. Higher educational level, higher income, employment and greater pre-pregnancy PA are reported to be associated with higher PA level [26, 27], while multiparity and unpleasant pregnancy symptoms are associated with less PA [28]. The influence of body mass index (BMI) on PA is mixed [28, 29].

Chinese families attach high importance to pregnant women and their foetuses and adhere to the traditional philosophy of protecting the foetus from miscarriage by advising pregnant women to rest as much as possible [30]. Despite this tradition, young women are accepting of modern healthcare-based recommendations and actively engage in PA. PA among Chinese women is sparsely studied. The proportion of pregnant women meeting PA recommendations varies from 11% in Tianjin [24] to 57.1% in Chengdu [25]. However, these studies are limited by their cross-sectional natures and represent only one city or district of China [24, 25].

Therefore, our study aims to investigate PA changes between early and mid-to-late pregnancy and the association of population characteristics with PA changes among Chinese women. Our study was based on the Chinese Pregnant Women Cohort Study-Peking Union Medical College (CPWCS-PUMC). The CPWCS-PUMC is a multicentre prospective observational study designed to investigate the lifestyles of pregnant Chinese women and the associations between lifestyle and obstetric or neonatal outcomes.

## Methods

### Study design

Our PA study was a longitudinal study using convenience sampling to recruit pregnant women who received early pregnancy evaluation within a certain month from July 2017 to November 2018 in 14 maternal and child healthcare hospitals and 10 academic hospitals located in 15 provinces of China. The location of the study recruitment hospital has been published previously [31]. All the 24 hospitals were public hospitals, and the cost of perinatal health care was largely covered by the government maternity insurance program and partly by individuals. Population characteristics that are biologically plausibility or historically reported to be associated with PA were considered as determinants investigated in our study. Detailed interviews were conducted at the initial recruitment clinic visit in early pregnancy to collect the population characteristics. Participants were asked to attend PA level assessment twice, with the first conducted in early pregnancy at the initial recruitment clinic visit and the second conducted in mid-to-late pregnancy at a prenatal clinic visit after 24 weeks of gestation.

### Study population

The inclusion criteria of the study population were as follows: (1) age 16 years or above, (2) pregnancy $\le$ 12 weeks, as estimated based on the last menstrual period; (3) permanent resident of the study recruitment district; (4) regular antenatal inspection with the intention of delivering in the study recruitment hospital; and (5) capable of online completion of the PA assessment. The exclusion criteria were as follows: (1) serious chronic diseases such as heart failure, pulmonary hypertension, restrictive lung disease, chronic renal disease, autoimmune disease, epilepsy, malignant tumors or other diseases which would restrict PA during pregnancy; and (2) multiple pregnancy. Written informed consent was obtained from all participants, and the study was approved by the ethics review committee of Peking Union Medical College (HS-1345).

**Fig. 1:**
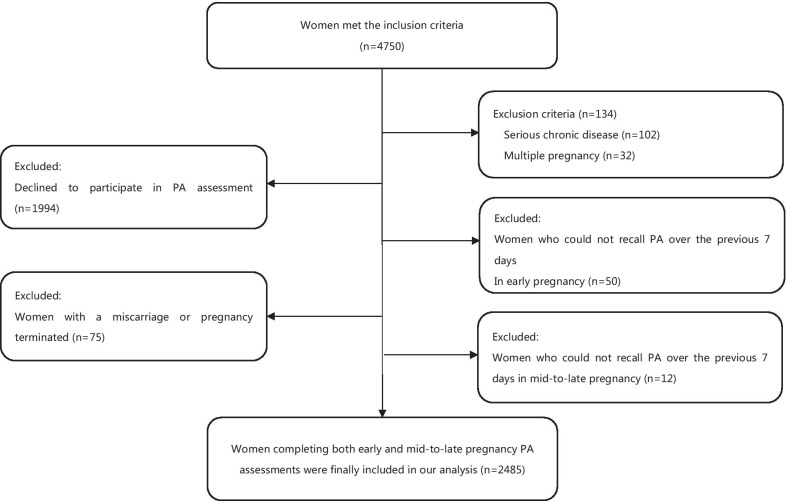
Flow chart of the study

Among 4750 women meeting the inclusion criteria of our PA study, 102 were excluded due to serious chronic diseases and 32 due to multiple gestation. A total of 1,994 declined to participate. Fifty participants could not recall their PA over the previous 7 days at the first PA assessment. A total of 2572 women completed the first PA assessment in early pregnancy. Seventy-five had a miscarriage or pregnancy termination between the two assessments. Twelve participants could not recall PA at the second PA assessment. A total of 2485 women with both PA information in early and mid-to-late pregnancy were finally included in the data analysis of the present study (Fig. 1).

### Measurements

#### Assessment of physical activity

PA was assessed in early and mid-to-late pregnancy using the International Physical Activity Questionnaire short form (IPAQ-SF) validated for the Chinese population [32, 33]. The IPAQ addresses three types of PA: high-intensity PA, medium-intensity PA, and walking. High-intensity PA refers to activities that require hard physical effort and that make breathing much harder than normal, such as heavy lifting, digging, or aerobics [34]. Medium-intensity PA refers to activities that take moderate physical effort and make breathing somewhat harder than normal, such as carrying light loads, bicycling at a regular pace, or table tennis [34]. Walking includes all walking for occupation, transportation, household, exercise and leisure. The frequency (days) and duration (minutes) of each PA over the previous seven days were investigated. Total energy expenditure (TEE) on PA per week was calculated as a total of three types of PA reported in the MET value × minutes per week. The values of 3.3, 4.0 and 8.0 were assigned to represent the MET values of walking, medium-intensity PA and high-intensity PA, respectively [35].

According to the IPAQ-SF, TEE on PA ≥ 600 MET min/week is defined as “moderate level” [35], and WHO recommends a minimum of 600 MET min/week PA to realize a health benefit [17]. Therefore, we defined PA with TEE ≥ 600 MET min/week as sufficient PA and PA with TEE < 600 MET min/week as insufficient PA. Sufficient PA in early pregnancy and insufficient PA in mid-to-late pregnancy indicated decreasing PA. Insufficient PA in early pregnancy and sufficient PA in mid-to-late pregnancy indicated increasing PA.

#### Determinants

Determinants included demographic, pregnancy and health characteristics. Demographic characteristics included age, residential region, ethnicity, educational level, annual household income and occupation. Pregnancy characteristics included parity and pregnancy intention. Health characteristics included prepregnancy BMI and history of smoking or drinking.

Age, ethnicity and pregnancy intention were considered to be biologically plausible determinants of PA. Educational level, income, occupation, parity, BMI and smoking were determinants which were historically reported to be associated with PA [26–29, 36].

Residential region, which was classified into East, Central and West China by economic development according to the Chinese Health Statistics Yearbook, was considered to be a plausible determinant of PA. East China was most urbanized and industrialized, while West China was most rural and agrarian. East China was considered to be the region with fastest economic growth, followed by Central and West China. Seven, nine and eight recruitment hospitals were located in East, Central, and West China, respectively.

The pregnancy was defined as an intended pregnancy if the couple had an intention to conceive. The pregnancy was defined as an unintended pregnancy if it was conceived accidentally. The prepregnancy BMI (kg/m2) was calculated based on the self-reported prepregnancy weight in kilograms and height in centimetres. BMI was categorized as underweight, normal weight, overweight and obese (< 18.5, 18.5–23.9, ≥ 24, respectively) [37, 38]. Smoking or drinking any type of alcohol over the previous 30 days when being surveyed was defined as a history of smoking or drinking.

### Statistical analysis

The population characteristics of all women included in the study are described. Categorical data are expressed as frequencies and percentages. Continuous data are expressed as means, standard deviations (SDs), medians and interquartile ranges (IQRs). TEE on PA, energy expenditure on each type of PA, and the proportion of energy expenditure on each type of PA to TEE on PA were compared between early and mid-to-late pregnancy using the Wilcoxon signed-rank test. The proportions of women with sufficient PA levels were compared between early and mid-to-late pregnancy using McNemar’s test. Multivariable logistic regression was used to calculate the odds ratios (OR) and 95% confidence intervals (CI) to address the following: (1) associations between population characteristics and sufficient PA in mid-to-late pregnancy among all women included in the study, (2) associations between population characteristics and increasing PA among the subset of women with insufficient PA levels in early pregnancy, and (3) associations between population characteristics and decreasing PA among the subset of women with sufficient PA levels in early pregnancy. *P* values < 0.05 were considered statistically significant. SPSS 22.0 (IBM, Armonk, NY, USA) was used for statistical analysis.

## Results

### Population characteristics

Data on population characteristics were obtained at the recruitment clinic visit at a mean gestational age of 10 weeks, ranging from 5 to 13. The characteristics of 2485 women who had both PA information in early and mid-to-late pregnancy and 2056 women who failed to complete the PA assessment (with 1994 declining to participate and 62 being unable to recall PA) are compared in Table 1. Compared with those failing to complete the PA assessment, women completing the PA assessment were more likely to be located in East China or have a university education or above, a higher medium income, a manual occupation, a nulliparous status, or an intended pregnancy (Table 1). The population characteristics by residential region are shown in Additional file 1: Table S1. There were significant differences in age, ethnicity, educational level, annual household income, occupation, parity and history of drinking between the three regional groups.

**Table 1:** Comparison of population characteristics between women who completed and failed to complete the PA assessment

Characteristics	Women who completed both PA assessments in early and mid-to-late pregnancy (n = 2585)	Women failed to complete the PA assessment (n = 2056)	*P* value
*Demographic characteristics*			
Age (years)			0.058
< 25	314 (12.64)	299 (14.27)	
25–29	1274 (51.27)	991 (48.20)	
30–34	638 (25.67)	568 (27.63)	
≥ 35	259 (10.42)	198 (9.63)	
Residential region			< 0.001^*a^
East	936 (37.67)	556(27.04)	
Central	770 (30.99)	795 (38.67)	
West	779 (31.35)	705 (34.29)	
Ethnicity			0.213
Han	2345 (94.37)	1922 (93.48)	
Minority	140 (5.63)	134 (6.52)	
Educational level			** < 0.001**
High school or below	700 (28.17)	757 (36.82)	
University or above	1785 (71.83)	1299 (63.18)	
Annual household income (RMB Yuan)			0.003^*b^
Low income (< 80,000)	553 (22.25)	518 (25.19)	
Lower medium income (80,000–109,999)	679 (27.32)	608 (29.57)	
Higher medium income (110,000–199,999)	485 (19.52)	333(16.20)	
High income (> 200,000)	768 (30.91)	597 (29.04)	
Occupation			< 0.001^*c^
Unemployed	592 (23.82)	548 (26.68)	
Manual occupation	1375 (55.33)	1001 (48.73)	
Non-manual occupation	518 (20.85)	505 (24.59)	
*Pregnancy characteristics*			
Parity			** < 0.001**
Nulliparity	1517 (61.05)	1109 (53.94)	
Multiparity	968 (38.95)	947 (46.06)	
Pregnancy intention			** < 0.001**
Intended	1849 (74.41)	1406 (68.39)	
Unintended	636 (25.59)	650 (31.61)	
*Health characteristics*			
Pre-pregnancy BMI (kg/m^2^)			0.855
< 18.5	325 (13.08)	275 (13.38)	
18.5–23.9	1595 (64.19)	1327 (64.54)	
≥ 24	565 (22.74)	454 (22.08)	
History of smoking			0.334
No	2424 (97.55)	1996 (97.08)	
Yes	61 (2.45)	60 (2.92)	
History of drinking			0.088
No	2356 (94.81)	1925 (93.63)	
Yes	129 (5.19)	131 (6.37)	

The chi-square test was used to compare the population characteristics between women who completed both PA assessments and those who failed to complete the PA assessment. A *P* value < 0.05 was considered significant, and significant values are marked with bold text

^*^Bonferroni correction was applied for multiple testing

^a^Women who completed the PA assessment were more likely to be located in East compared with Central (*P* < *0.001*) or West China (*P* < *0.001*)

^b^Women who completed the PA assessment were more likely to have a higher medium income than a lower medium income (*P* = *0.001*) or a low income (*P* = *0.003*)

^c^Women who completed the PA assessment were more likely to be employed with a manual occupation than unemployed (*P* = *0.001*) or employed with a non-manual occupation (*P* < 0.001)

### Change in PA across pregnancy

**Fig. 2:**
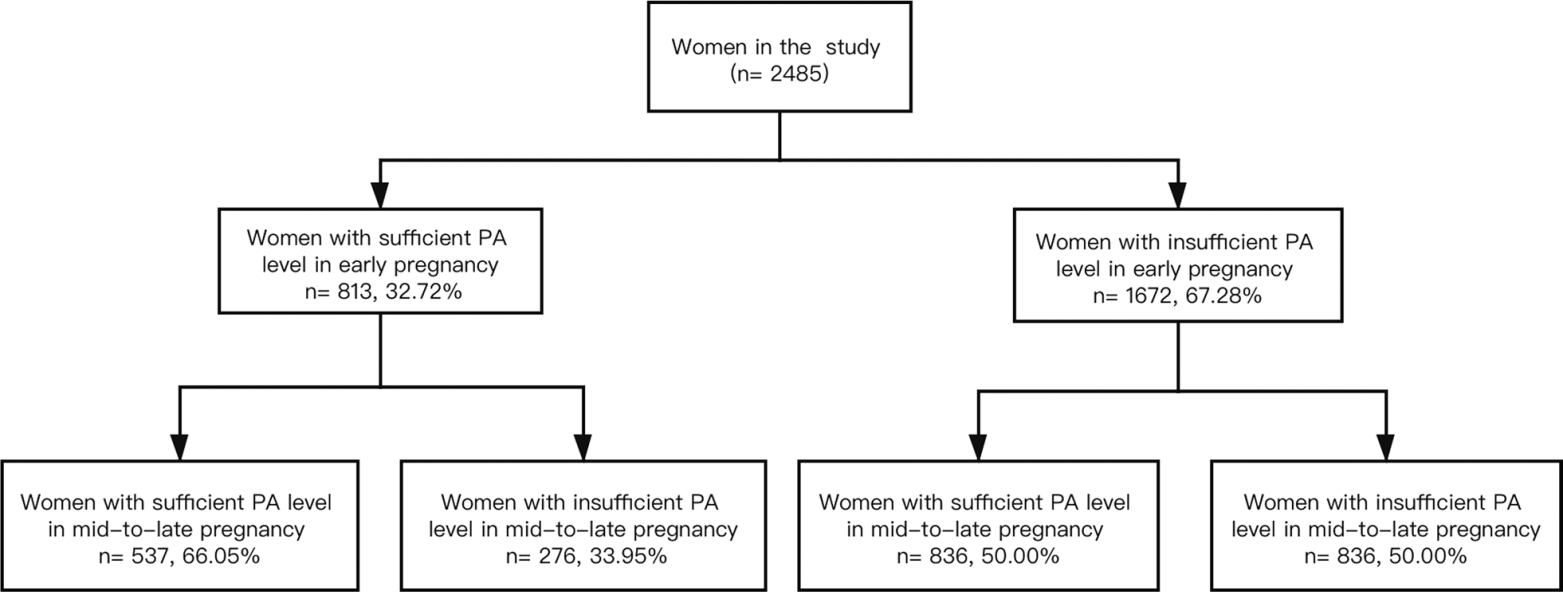
The proportions of women with sufficient and insufficient PA levels in early and mid-to-late pregnancy

PA was assessed in early pregnancy at a mean gestational age of 10 weeks, ranging from 5 to 13, and reassessed in mid-to-late pregnancy at a mean gestational age of 32 weeks, ranging from 24 to 40. Table 2 shows that TEE on PA and energy expenditure on each type of PA increased significantly from early to mid-to-late pregnancy (*P* < 0.001). The largest proportion of PA across pregnancy comprised walking (median, 100%). The proportion of women with sufficient PA levels also increased significantly from 32.72% to 55.25% from early to mid-to-late pregnancy (*P* < 0.001). Among 1672 women with insufficient PA levels in early pregnancy, 836 (50.00%) remained insufficient, and 836 (50.00%) had increasing PA. Among 813 women with sufficient PA levels in early pregnancy, 537 (66.05%) maintained sufficient PA, and 276 (33.95%) had decreasing PA (see Fig. 2).

**Table 2:** Comparison of PA between early and mid-to-late pregnancy among Chinese pregnant women (n = 2485)

PA indicators	Early pregnancy		Mid-to-late pregnancy		*P* value
	Mean (SD)	Median (IQR)	Mean (SD)	Median (IQR)	
TEE on PA (MET min/week)	791.93 (1057.56)	396.00 (66.00, 1152.00)	1193.14 (1283.51)	813.00 (356.40, 1411.00)	< 0.001
High-intensity PA					
Energy expenditure (MET min/week)	43.24 (327.85)	0.00 (0.00, 0.00)	86.16 (506.34)	0.00 (0.00, 0.00)	< 0.001
Proportion due to TEE on PA (%)	2.46 (11.79)	0.00 (0.00, 0.00)	2.96 (12.54)	0.00 (0.00, 0.00)	< 0.001
Medium-intensity PA					
Energy expenditure (MET min/week)	73.02 (316.18)	0.00 (0.00, 0.00)	124.45 (429.27)	0.00 (0.00, 0.00)	< 0.001
Proportion due to TEE on PA (%)	5.39 (16.04)	0.00 (0.00, 0.00)	7.03 (17.09)	0.00 (0.00, 0.00)	< 0.001
Walking					
Energy expenditure (MET min/week)	675.66 (862.19)	396.00 (49.50, 990,00)	982.53 (929.47)	693.00 (297.00, 1386.00)	< 0.001
Proportion due to TEE on PA (%)	92.15 (20.83)	100.00 (100.00, 100.00)	90.00 (22.06)	100.00 (100.00, 100.00)	< 0.001

The Wilcoxon signed-rank test was used to compare PA indicators between early and mid-to-late pregnancy. A *P* value < 0.05 was considered significant

### Association of population characteristics and PA change

Table 3 shows that women in West China (OR 1.247, 95% CI 1.012–1.537; *P* = 0.038) were most likely and that women in Central China (OR 0.747, 95% CI 0.609–0.916; *P* = 0.005) were least likely to have sufficient PA in mid-to-late pregnancy. Women with a smoking history (OR 0.551, 95% CI 0.315–0.964; *P* = 0.037) were less likely, and women with sufficient PA levels in early pregnancy (OR 1.897, 95% CI 1.583–2.274; *P* < 0.001) were more likely to have sufficient PA in mid-to-late pregnancy. Table 4 shows that in the subset of women with insufficient PA levels in early pregnancy, women in West China (OR 1.387, 95% CI 1.078–1.783; *p* = 0.011) were most likely, and women in Central China (OR 0.721, 95% CI 0.562–0.925; *P* = 0.010) were least likely to increase PA across their pregnancies. Women with a smoking history (OR 0.480, 95% CI 0.242–0.955; *P* = 0.036) were less likely to increase PA across their pregnancies. In the subset of women with sufficient PA levels in early pregnancy, women with educational levels of university or above (OR 0.662, 95% CI 0.442–0.991; *P* = 0.045) were less likely to decrease PA across their pregnancies.

**Table 3:** Associations between population characteristics and sufficient PA in mid-to-late pregnancy

Characteristics	Sufficient PA in mid-to-late pregnancy (n = 1373)		*P* value
	N (%)	OR (95%CI)	
*Demographic characteristics*			
Age (years)			
< 25	158 (11.51)	1.000	
25–29	730 (53.17)	1.212 (0.922, 1.592)	0.168
30–34	342 (24.91)	1.068 (0.783, 1.457)	0.677
≥ 35	143 (10.42)	1.186 (0.808, 1.743)	0.384
*Residential region*			
East	527 (38.38)	1.000	
Central	368 (26.80)	0.747 (0.609, 0.916)	**0.005**
West	478 (34.81)	1.247 (1.012, 1.537)	**0.038**
*Ethnicity*			
Han	1296 (94.39)	1.000	
Minority	77 (5.61)	0.976 (0.677, 1.408)	0.898
Educational level			
High school or below	371 (27.02)	1.000	
University or above	1002 (72.98)	1.023 (0.818, 1.279)	0.842
*Annual household income (RMB Yuan)*			
Low income (< 80,000)	295 (21.49)	1.000	
Lower medium income (80,000–109,999)	359 (26.15)	0.950 (0.745, 1.211)	0.680
Higher medium income (110,000–199,999)	288 (20.98)	1.185 (0.901, 1.557)	0.224
High income (> 200,000)	431 (31.39)	1.096 (0.853, 1.409)	0.472
*Occupation*			
Unemployed	316 (23.02)	1.000	
Manual occupation	756 (55.06)	0.888 (0.702, 1.124)	0.324
Non-manual occupation	301 (21.92)	1.089 (0.838, 1.415)	0.525
*Pregnancy characteristics*			
Parity			
Nulliparity	856 (62,35)	1.000	
Multiparity	517 (37.65)	0.926 (0.759, 1.131)	0.452
*Pregnancy intention*			
Intended	1038 (75.60)	1.000	
Unintended	335 (24.40)	0.938 (0.773, 1.139)	0.520
*Health characteristics*			
Prepregnancy BMI (kg/m2)			
< 18.5	183 (13.33)	1.069 (0.829, 1.377)	
18.5–23.9	883 (64.31)	1.000	0.608
≥ 24	307 (22.36)	1.035 (0.842, 1.272)	0.746
*History of smoking*			
No	1349 (98.25)	1.000	
Yes	24 (1.75)	0.551 (0.315, 0.964)	**0.037**
*History of drinking*			
No	1304 (94.97)	1.000	
Yes	69 (5.03)	1.046 (0.714, 1.531)	0.819
*PA level in early pregnancy*			
Insufficient	836 (60.89)	1.000	
Sufficient	537 (39.11)	1.897 (1.583, 2.274)	** < 0.001**

OR was adjusted for the rest of the variables in the table. A *P* value < 0.05 was considered significant, and significant values are marked with bold text

**Table 4:** Associations between population characteristics and increasing or decreasing PA across pregnancy

Characteristics	Insufficient PA in early pregnancy (n = 1672)		OR (95% CI)	*P* value	Sufficient PA in early pregnancy (n = 813)		OR (95% CI)	*P* value
	Increasing PA (n = 836)	Maintaining insufficient PA (n = 836)			Decreasing PA (n = 276)	Maintaining sufficient PA (n = 537)		
	n (%)	n (%)			n (%)	n (%)		
*Demographic characteristics*								
Age (years)								
< 25	103 (12.32)	122 (14.59)	1.000		34(12.32)	55(10.24)	1.000	
25–29	442 (52.87)	405 (48.44)	1.251 (0.904, 1.731)	0.178	139 (50.36)	288(53.63)	0.911 (0.542, 1.530)	0.724
30–34	204 (24.40)	218 (26.08)	1.105 (0.763, 1.601)	0.598	78 (28.26)	138 (25.70)	1.103 (0.615, 1.978)	0.742
≥ 35	87 (10.41)	91 (10.89)	1.107 (0.699, 1.754)	0.664	25 (9.06)	56 (10.43)	0.775 (0.375, 1.601)	0.491
*Residential region*								
East	309 (36.96)	309 (36.96)	1.000		100 (36.23)	218 (40.60)	1.000	
Central	215 (25.72)	308 (36.84)	0.721 (0.562, 0.925)	**0.010**	94 (34.06)	153 (28.49)	1.274 (0.880, 1.844)	0.199
West	312 (37.32)	219 (26.20)	1.387 (1.078, 1.783)	**0.011**	82 (29.71)	166 (30.91)	1.048 (0.716, 1.532)	0.811
Ethnicity								
Han	788 (94.26)	790 (94.50)	1.000		259 (93.84)	508 (94.60)	1.000	
Minority	48 (5.74)	46 (5.50)	0.944 (0.606, 1.471)	0.799	17 (6.16)	29 (5.40)	1.084 (0.558, 2.106)	0.811
Educational level								
Senior school or below	244 (29.19)	245 (29.31)	1.000		84 (30.43)	127(23.65)	1.000	
University or above	592 (70.81)	591 (70.69)	0.879 (0.671, 1.150)	0.348	192 (69.57)	410(76.35)	0.662 (0.442, 0.991)	**0.045**
*Annual household income (RMB Yuan)*								
Low income (< 80,000)	183 (21.89)	196 (23.44)	1.000		62 (22.46)	112(20.86)	1.000	
Lower medium income (80,000–109,999)	226 (27.03)	239 (28.59)	1.017 (0.759, 1.363)	0.910	81 (29.35)	133(24.77)	1.262 (0.806, 1.978)	0.305
Higher medium income (110,000–199,999)	178 (21.29)	142 (16.99)	1.345 (0.964, 1.875)	0.081	55 (19.93)	110(20.48)	1.086 (0.669, 1.763)	0.740
High income (> 200,000)	249 (29.78)	259 (30.98)	1.087 (0.801, 1.474)	0.593	78 (28.26)	182(33.89)	0.896 (0.565, 1.419)	0.639
Occupation								
Unemployed	206 (24.64)	217 (25.96)	1.000		59 (21.38)	110(20.48)	1.000	
Manual occupation	446 (53.35)	453 (54.19)	0.926 (0.696, 1.232)	0.597	166 (60.14)	310(57.73)	1.221 (0.795, 1.876)	0.362
Non-manual occupation	184 (22.01)	166 (19.86)	1.104 (0.809, 1.505)	0.533	51 (18.48)	117(21.79)	0.978 (0.592, 1.616)	0.931
Pregnancy characteristics								
Parity								
Nulliparity	527 (63.04)	493 (58.07)	1.000		168 (60.87)	330(61.45)	1.000	
Multiparity	309 (36.96)	343 (41.03)	0.881 (0.694, 1.118)	0.297	108 (39.13)	207(38.55)	0.888 (0.613, 1.288)	0.532
Pregnancy intention								
Intended	629 (75.24)	614 (73.44)	1.000		197 (71.38)	409(76.16)	1.000	
Unintended	207 (24.76)	222 (26.56)	0.988 (0.781, 1.250)	0.921	79 (28.62)	128(23.84)	1.218 (0.860, 1.725)	0.266
Health characteristics								
Pre-pregnancy BMI (kg/m^2^)								
< 18.5	103 (12.32)	111 (13.28)	0.953 (0.701, 1.297)	0.761	31 (11.23)	80(14.90)	1.709 (0.439, 1.143)	0.158
18.5–23.9	534 (63.88)	532 (63.64)	1.000		180 (65.22)	349(64.99)	1.000	
≥ 24	199 (23.80)	193 (23.09)	1.123 (0.877, 1.437)	0.359	65 (23.55)	108(20.11)	1.152 (0.787, 1.687)	0.467
History of smoking								
No	821 (98.21)	806 (96.41)	1.000		269 (97.46)	528(98.32)	1.000	
Yes	15 (1.79)	30 (3.59)	0.480 (0.242, 0.955)	**0.036**	7 (2.54)	9(1.68)	1.446 (0.515, 4.061)	0.484
History of drinking								
No	791 (94.62)	784 (93.78)	1.000		268 (97.10)	513(95.53)	1.000	
Yes	45 (5.38)	52 (6.22)	0.917 (0.590, 1.427)	0.701	8 (2.90)	24(4.47)	0.615 (0.267, 1.416)	0.253

OR was adjusted for the rest of the variables in the table. A *P* value < 0.05 was considered significant, and significant values are marked with bold text

## Discussion

To our knowledge, this is the first multicentre longitudinal cohort study to investigate changes in PA across pregnancy in a Chinese population. We found that PA levels increased from early to mid-to-late pregnancy and that more than half of the women eventually had sufficient PA as recommended. Walking was the dominant form of PA. Women with sufficient PA levels in early pregnancy were more likely to maintain or achieve sufficient PA across their pregnancies. PA levels varied in different regions of China, with women in the West being most likely and those in the Central being least likely to have sufficient and increasing PA. Habitual smoking was inversely associated with sufficient and increasing PA. Women with higher educational levels were less likely to decrease PA across pregnancy.

Our study found that the proportion of pregnant women achieving the recommended PA level increased from 32.72% in early pregnancy to 55.25% in mid-to-late pregnancy. Studies investigating PA levels at different pregnancy stages are limited. In contrast to our study, studies of western populations showed that PA decreased or remained unchanged as pregnancy progressed [39, 40]. Regarding studies with Chinese populations, the proportion of women achieving the recommended level remained at a low level of 11% across the pregnancies of urban women from Tianjin [24] and increased from 53.8% in the first trimester to 61.4% in the third trimester among women from Chengdu [25]. But both of these studies were cross-sectional, and the changes in PA were concluded from different subsets of women. Our study was a multicentre study, and PA was surveyed in the same sample of women longitudinally at different pregnancy stages, resulting in a better representation of the Chinese population and in less bias. However, women who declined to participate in the PA assessment or could not recall PA level when being surveyed were not included in our final analysis. We found that women who completed the two PA assessments were more likely to have the characteristics that were reported to be positively associated with PA, such as a higher educational level. There might be a potential bias in that the women included in the study were those with a higher PA level. This might be part of the reason why our population had increasing PA across pregnancy. As the pregnancy progressed, the fear of miscarriage and the unpleasant pregnancy symptoms were gradually alleviated, which might also contribute to the resume of PA in mid-to-late pregnancy.

Our findings were consistent with those of one previous study using the IPAQ-SF, namely, that PA with medium or higher intensity contributed less to TEE during pregnancy [22]. Our study found that walking was the dominant form of PA. In this study, walking included all walking, namely, walking related to occupation, transportation, the household, exercise and leisure. Walking is the form of moderate-intensity PA indicated by the WHO recommendation and the form of exercise recommended by the American College of Obstetricians and Gynaecologist (ACOG) during pregnancy [1, 2, 17]. Chinese culture holds tight to traditional concepts of not walking fast, not running and not jumping during pregnancy, but walking is not restricted [30]. Therefore, it may be more reasonable and easier for prenatal healthcare providers to encourage women to walk appropriately to meet the sufficient level of 600 MET min/week than to persuade them to participate in other forms of PA.

It has been well documented that prepregnancy PA habits are strongly associated with PA levels during pregnancy [39, 40]. Our study found that women with sufficient PA levels at baseline were more likely to maintain or achieve sufficient PA across their pregnancies, further validating the fact that a good lifestyle is beneficial in the long term.

There were significant differences in the population characteristics among the three regional groups. Women in West China were more likely to have features favouring PA, such as a higher educational level, employment with a manual occupation and nulliparity, which may explain why they were more likely to have sufficient and increasing PA. The higher proportion of individuals of non-Han ethnicity may also play a role. Our results were consistent with one previous study reporting high and increasing PA across pregnancy in Chengdu, which located in West China [25]. One study conducted in Tianjin, which located in East China, found low and unchanged PA across pregnancy [24]. However, their study was limited by having only urban participants in one city. More studies are required to clarify the regional difference in PA.

Among the other correlates investigated, a history of smoking was inversely associated with sufficient and increasing PA. The findings were consistent with other study data showing that smokers had a more sedentary lifestyle [36]. The combined risk of smoking and low PA levels may make women more vulnerable to adverse pregnancy outcomes.

A recent systemic review revealed that younger age, higher educational level, higher income, employment, nulliparity, and normal weight were positively associated with PA during pregnancy, but the correlations were weak [39]. However, our study found that except for educational level, these factors were not associated with changes in PA across pregnancy. In our study, women with higher educational level tended to maintain sufficient PA throughout the whole pregnancy.

There were several limitations in our study. First, the study might have bias due to the self-report measure used to assess PA. Second, the generalization of our results may be limited to convenience sampling. Third, our study population may have a higher PA level since only those who agreed to participate and could recall PA were finally included in the analysis. Fourth, the large time frame within which PA was surveyed (especially the second survey) was partly responsible for the large range of PA level. The difference in the time interval between two PA assessments may have an influence on the result of the PA change across pregnancy. Finally, our study did not include pre-pregnancy PA level, discomfort during pregnancy and subjective factors, such as self-efficacy or perceived behavioural control, which might influence PA [29, 41]. Further analyses that include these factors as determinants of PA are needed.

## Conclusions

To our knowledge, our study is the first multicentre longitudinal cohort study to investigate changes in PA across pregnancy among Chinese women. Our findings indicated that PA increased as pregnancy progressed, and that walking was the dominant form of PA. Further research is needed to better understand correlates of PA change and develop appropriate interventions for PA to improve maternal health among Chinese women.

## Supplementary Information

**Additional file 1**. Table S1. Population characteristics by residential region.

## Data Availability

The datasets used and/or analysed during the current study are available from the corresponding author on reasonable request.

## Abbreviations

ACOG: American College of Obstetricians and Gynecologists
BMI: Body mass index
CI: Confidence interval
CPWCS-PUMC: Chinese Pregnant Women Cohort Study-Peking Union Medical College
IPAQ-SF: International Physical Activity Questionnaire short form
IQR: Interquartile range
MET: Metabolic equivalent task
OR: Odds ratio
PA: Physical activity
SD: Standard deviation
TEE: Total energy expenditure
WHO: World Health Organization
