# Supplementary material for: Changes in physical activity across pregnancy among Chinese women: a longitudinal cohort study
Source: BMC Womens Health. 2021 Jun 6;21:236. doi: 10.1186/s12905-021-01377-3 (PMC8183053; doi:10.1186/s12905-021-01377-3)
Supplement: Supplementary file 1 — Additional file 1. Table S1. Population characteristics by residential region. [file 12905_2021_1377_MOESM1_ESM.docx]

**Supplementary Table 1.** Population characteristics by residential region

| Characteristics | Total | East | Central | West | *P* value |
| --- | --- | --- | --- | --- | --- |
| Demographic characteristics |  |  |  |  |  |
| Age (years) |  |  |  |  | **0.021*** |
| <25 | 314 (12.64) | 116 (12.39) | 121 (15.71) | 77 (9.88) |  |
| 25-29 | 1274 (51.27) | 470 (50.21) | 393 (51.04) | 411 (52.76) |  |
| 30-34 | 638 (25.67) | 255 (27.24) | 183 (23.77) | 200 (25.67) |  |
| ≥35 | 259 (10.42) | 95 (10.15) | 73 (9.48) | 91 (11.68) |  |
| Ethnicity |  |  |  |  | **<0.001*** |
| Han | 2345 (94.37) | 907 (96.90) | 746 (96.88) | 692 (88.83) |  |
| Minority | 140 (5.63) | 29 (3.10) | 24 (3.12) | 87 (11.17) |  |
| Educational level |  |  |  |  | **<0.001*** |
| High school or below | 700 (28.17) | 289 (30.88) | 240 (31.17) | 171 (21.95) |  |
| University or above | 1785 (71.83) | 647 (69.12) | 530 (68.83) | 608 (78.05) |  |
| Annual household income (RMB Yuan) |  |  |  |  |  |
| Low income (<80,000) | 553 (22.25) | 181 (19.34) | 215 (27.92) | 157 (20.15) | **<0.001*** |
| Lower medium income (80,000-109,999) | 679 (27.32) | 241 (25.75) | 218 (28.31) | 220 (28.24) |  |
| Higher medium income (110,000-199,999) | 485 (19.52) | 180 (19.23) | 128 (16.62) | 177 (22.72) |  |
| High income (>200,000) | 768 (30.91) | 334 (35.68) | 209 (27.14) | 225 (28.88) |  |
| Occupation |  |  |  |  | **<0.001*** |
| Unemployed | 592 (23.82) | 186 (19.87) | 247 (32.08) | 159 (20.41) |  |
| Manual occupation | 1375 (55.33) | 510 (54.49) | 395 (51.30) | 470 (60.33) |  |
| Non-manual occupation | 518 (20.85) | 240 (25.64) | 128 (16.62) | 150 (19.26) |  |
| Pregnancy characteristics |  |  |  |  |  |
| Parity |  |  |  |  | **<0.001*** |
| Nulliparity | 1517 (61.05) | 528 (55.81) | 447 (58.05) | 542 (70.21) |  |
| Multiparity | 968 (38.95) | 415 (44.19) | 323(41.95) | 230 (29.79) |  |
| Pregnancy Intention |  |  |  |  | 0.364 |
| Intended | 1849 (74.41) | 710 (75.85) | 561(72.86) | 578 (74.20) |  |
| Unintended | 636 (25.59) | 226 (24.15) | 209 (27.14) | 201 (25.80) |  |
| Health characteristics |  |  |  |  |  |
| Pre-pregnancy BMI (kg/m^2^) |  |  |  |  | 0.415 |
| <18.5 | 325 (13.08) | 115 (12.29) | 108 (14.03) | 102 (13.09) |  |
| 18.5-23.9 | 1595 (64.19) | 615(65.71) | 473 (61.43) | 507 (65.08) |  |
| ≥24 | 565 (22.74) | 206 (22.01) | 189 (24.55) | 170 (21.82) |  |
| History of smoking |  |  |  |  | 0.515 |
| No | 2424 (97.55) | 915 (97.76) | 747(97.01) | 762 (97.82) |  |
| Yes | 61 (2.45) | 21 (2.24) | 23 (2.99) | 17(2.18) |  |
| History of drinking |  |  |  |  | **0.004*** |
| No | 2356 (94.81) | 880 (94.02) | 747 (97.01) | 729 (93.58) |  |
| Yes | 129 (5.19) | 56 (5.98) | 23 (2.99) | 50 (6.42) |  |

Note: The chi-square test was used to compare population characteristics between the three reginal groups, *P* value <0.05 was considered significant, and significant values are marked with bold text.

* Bonferroni correction was applied for the multiple testing among 3 regions. Compared with women in Central China, women in West China had a higher proportion of individuals with an age >25 (*P* <0.023), non-Han ethnicity (*P* <0.005), higher educational level (*P* <0.001), higher medium income (*P* <0.001) and high income *(P* =0.006), a manual occupation (*P* <0.001) and a non-manual occupation (*P* <0.001), nulliparity (*P* <0.001) and history of drinking (*P* =0.001). Compared with the East China participants, the group in West China had a higher proportion of women of non-Han ethnicity (*P* <0.005), with a higher educational level (*P* <0.001), employed with a manual occupation (*P* =0.001) and with nulliparity (*P* <0.001).
